# Complete genome sequence of *Pedobacter heparinus* type strain (HIM 762-3^T^)

**DOI:** 10.4056/sigs.22138

**Published:** 2009-07-20

**Authors:** Cliff Han, Stefan Spring, Alla Lapidus, Tijana Glavina Del Rio, Hope Tice, Alex Copeland, Jan-Fang Cheng, Susan Lucas, Feng Chen, Matt Nolan, David Bruce, Lynne Goodwin, Sam Pitluck, Natalia Ivanova, Konstantinos Mavromatis, Natalia Mikhailova, Amrita Pati, Amy Chen, Krishna Palaniappan, Miriam Land, Loren Hauser, Yun-Juan Chang, Cynthia C. Jeffries, Elizabeth Saunders, Olga Chertkov, Thomas Brettin, Markus Göker, Manfred Rohde, Jim Bristow, Jonathan A. Eisen, Victor Markowitz, Philip Hugenholtz, Nikos C. Kyrpides, Hans-Peter Klenk, John C. Detter

**Affiliations:** 1 DOE Joint Genome Institute, Walnut Creek, California, USA; 2 Los Alamos National Laboratory, Bioscience Division, Los Alamos, New Mexico USA; 3 DSMZ - German Collection of Microorganisms and Cell Cultures GmbH, Braunschweig, Germany; 4 Biological Data Management and Technology Center, Lawrence Berkeley National Laboratory, Berkeley, California, USA; 5 Oak Ridge National Laboratory, Oak Ridge, Tennessee, USA; 6 HZI - Helmholtz Centre for Infection Research, Braunschweig, Germany; 7 University of California Davis Genome Center, Davis, California, USA

**Keywords:** mesophile, strictly aerobic, dry soil, Gram-negative, flexible rods, heparinase producer, *Sphingobacteriaceae*

## Abstract

*Pedobacter heparinus* (Payza and Korn 1956) Steyn *et al.* 1998 comb. nov. is the type species of the rapidly growing genus *Pedobacter* within the family *Sphingobacteriaceae* of the phylum ‘*Bacteroidetes’*. *P. heparinus* is of interest, because it was the first isolated strain shown to grow with heparin as sole carbon and nitrogen source and because it produces several enzymes involved in the degradation of mucopolysaccharides. All available data about this species are based on a sole strain that was isolated from dry soil. Here we describe the features of this organism, together with the complete genome sequence, and annotation. This is the first report on a complete genome sequence of a member of the genus *Pedobacter*, and the 5,167,383 bp long single replicon genome with its 4287 protein-coding and 54 RNA genes is part of the *** G****enomic* *** E****ncyclopedia of* *** B****acteria and* *** A****rchaea * project.

## Introduction

*Pedobacter heparinus* strain HIM 762-3 (DSM 2366 = ATCC 13125 = JCM 7457 and other culture collections) is the type strain of the species, and was first described in 1956 by Payza and Korn as *Flavobacterium heparinum* (basonym) [1]. The authors of the original species description provided no type strain designation when depositing their isolate in the American Type Culture Collection (ATCC 13125^T^). In the Approved Lists of Bacterial Names (1980) the type strain of *F heparinum* appears as ATCC 13125^T^. Strain HIM 762-3^T^ was deposited in the DSMZ culture collection by Walter Mannheim (Marburg) in 1982, and ATCC is using the same strain designation for their ATCC 13125^T^. Following successive transfers of this species to the genera *Cytophaga* [2] and *Sphingobacterium* [3] the present name *P. heparinus* was proposed by Steyn *et al.* in 1998 [4]. Enzymes produced by *P. heparinus* could be successfully used for the study of the structure of heparin and chondroitin, important animal mucopolysaccharides with sulfate groups. Here we present a summary classification and a set of features for *P. heparinus* HIM 762-3^T^ (Table 1), together with the description of the complete genomic sequencing and annotation.

**Table 1:** Classification and general features of *P. heparinus* HIM 762-3^T^ based on MIGS recommendations [5]

MIGS ID	Property	Term	Evidence code
	Current classification	Domain *Bacteria*	
		Phylum *Bacteroidetes*	
		Class *Sphingobacteria*	TAS [6]
		Order *Sphingobacteriales*	TAS [6]
		Family *Sphingobacteriaceae*	TAS [4]
		Genus *Pedobacter*	TAS [1]
		Species *Pedobacter heparinus*	TAS [1]
		Type strain HIM 762-3	
	Gram stain	negative	TAS [4]
	Cell shape	rod-shaped	TAS [4]
	Motility	probably gliding, non-flagellated	TAS [4]
	Sporulation	non-sporulating	TAS [4]
	Temperature range	mesophile, 10-35°C	TAS [2]
	Optimum temperature	25-30°C for growth	TAS [2]
	Salinity	0-3% NaCl	TAS [2]
MIGS-22	Oxygen requirement	aerobe	TAS [1,2]
	Carbon source	carbohydrates, glycosaminoglycans	TAS [1,4]
	Energy source	chemoorganotroph	TAS [1,2,4]
MIGS-6	Habitat	soil	TAS [1]
MIGS-15	Biotic relationship	free living	NAS
MIGS-14	Pathogenicity	none	NAS
	Biosafety level	1	TAS [7]
	Isolation	not reported	
MIGS-4	Geographic location	not reported	
MIGS-5	Sample collection time	before 1956	NAS
MIGS-4.1 MIGS-4.2	Latitude – Longitude	not reported	
MIGS-4.3	Depth	not reported	
MIGS-4.4	Altitude	not reported	

Evidence codes - IDA: Inferred from Direct Assay (first time in publication); TAS: Traceable Author Statement (i.e., a direct report exists in the literature); NAS: Non-traceable Author Statement (i.e., not directly observed for the living, isolated sample, but based on a generally accepted property for the species, or anecdotal evidence). These evidence codes are from the Gene Ontology project [8]. If the evidence code is IDA, then the property was directly observed for a live isolate by one of the authors or an expert mentioned in the acknowledgements.

## Classification and features

Until now the species *P. heparinus* has comprised only one strain, HIM 762-3^T^. Two closely related strains, Gsoil 042^T^ and LMG 10353^T^, were recently described and affiliated to the species *P. panaciterrae* [9] and *P. africanus* [4], respectively, based on low DNA-DNA binding values to the type strain of *P. heparinus*. Unclassified strains with significant (98%) 16S rRNA sequence similarity to these species were observed from Ginseng field soil (AM279216), dune grassland soil [10] and activated sludge samples [11]. Environmental genomic surveys indicated highly similar (96% 16S rRNA gene sequence identity) phylotypes in BAC libraries generated from *Brassica rapa* subsp*. pekinensis* (field mustard) and *Sorghum bicolor* (milo) (ED512136, DX082358, BZ779630). A draft genome sequence of the unclassified *Pedobacter* strain BAL39 isolated from the Baltic Sea was recently determined by the J. Craig Venter Institute (Genbank NZ_ABCM00000000).

**Figure 1:**
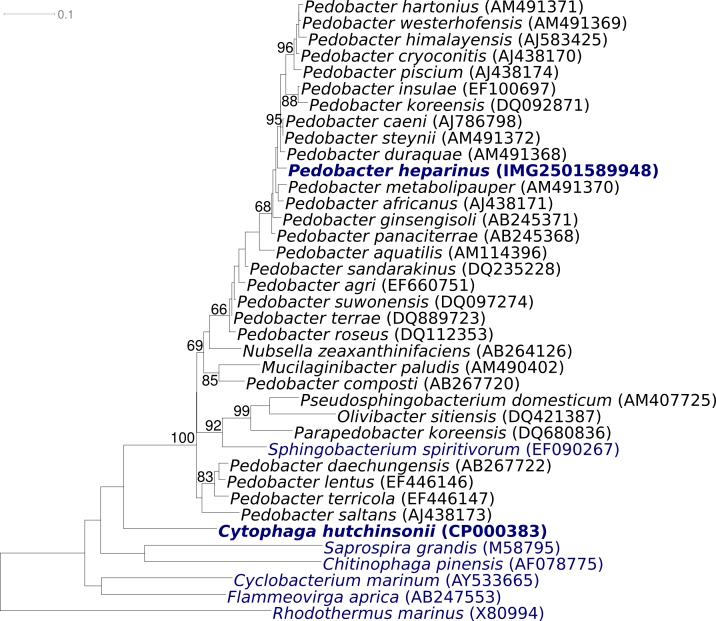
Phylogenetic tree of *P. heparinus* strain HIM 762-3^T^ and the type strains of the genus *Pedobacter*, as well as all type strains of the other genera within the family *Sphingobacteriaceae,* inferred from 1373 aligned characters [12,13] of the 16S rRNA gene under the maximum likelihood criterion [14]. The tree was rooted with the type strains of the other families within the order ‘*Sphingobacteriales’.* The branches are scaled in terms of the expected number of substitutions per site. Numbers above branches are support values from 1000 bootstrap replicates if larger than 60%. Lineages with type strain genome sequencing projects registered in GOLD [15] are shown in blue, published genomes in bold.

Figure 1 shows the phylogenetic neighborhood of *P. heparinus* strain HIM 762-3^T^ in a 16S rRNA based tree. The sequences of the three 16S rRNA gene copies in the genome are identical and differ by only one nucleotide from the previously published 16S rRNA gene sequence derived from DSM 2366 (AJ438172).

**Figure 2:**
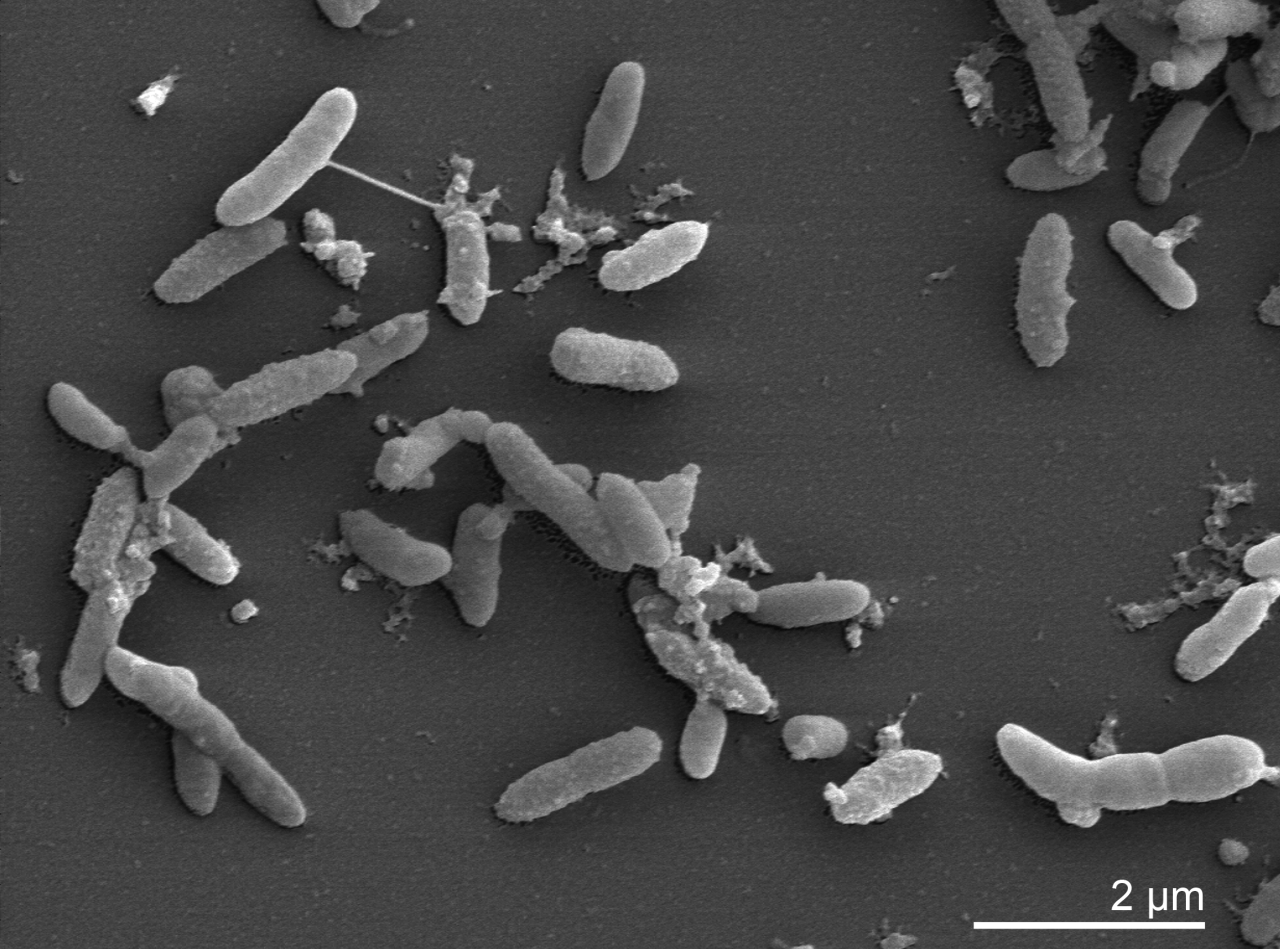
Scanning electron micrograph of *P. heparinus* HIM 762-3^T^

*P. heparinus* cells are Gram-negative, non-flagellated, non-spore-forming, flexible rods with rounded or slightly tapered ends. Cell width is 0.4-0.5 µm and cell length can vary from 0.7 to 6 µm. Protrusions can be observed on the cell surface (Figure 2). Some authors have reported a gliding motility [2]. Colonies are 1–4 mm in diameter and produce a yellowish, water insoluble, non-fluorescent pigment upon growth on nutrient agar [4]. Growth occurs at 10 and 35°C, but not above 37°C. The optimal temperature for growth is between 25 and 30°C [2]. The pH range for growth is 7-10 [2]. Strain HIM 762-3^T^ is strictly aerobic and prefers carbohydrates and sugars as carbon sources. Neither nitrate nor nitrite is reduced. The strain is catalase and oxidase positive. Acetoin is produced from pyruvate, but indole is not produced from tryptophan. HIM 762-3^T^ is negative for gelatinase, urease and DNase, but esculin and Tween 20–80 are hydrolyzed; acid and alkaline phosphatases are present [4]. The strain does not require vitamins, but L-histidine is essential for growth [16]. A special feature of strain HIM 762-3^T^ is its ability to degrade acidic sulfated mucoheteropolysaccharides, like heparin and chondroitin that are formed in various animal tissues. Enzymes involved in the degradation of heparin are only produced after induction by the substrate and are formed intracellularly [16]. Several different types of enzymes are involved in the complete degradation of heparin, including heparinases, glycuronidase, sulfoesterases and sulfamidases [17]. The first step in the degradation of heparin is catalyzed by heparinase (EC 4.2.2.7), an α1-4-eliminase which acts specifically on the glycosidic linkage between *N*-sulfated D-glucosamine and sulfated D-glucuronic acid (or L-iduronic acid). The use of heparinase in the elucidation of the structure of heparin, blood deheparinization or enzymatic assay of heparin have been proposed [16]. The genetics of heparin and chondrotitin degradation in *P. heparinus* was studied extensively and a high-level expression system for glycosaminoglycan lyases in this species has been developed [18]. Three different genes encoding heparinases (*hepA*-*C*) and two different genes for chondroitinases (*cslA* and *cslB*) could be characterized [18]. The crystal structures of the chondroitinase B [19] and heparinase II [20] of *P. heparinus* were resolved at high resolution.

### Chemotaxonomy

The peptidoglycan structure of strain HIM 762-3^T^ is still unknown. The cellular fatty acid pattern is dominated by saturated, iso branched and hydroxylated acids. The most abundant non-polar cellular fatty acids are *iso*-15:0, 16:1 ω7*c*, *iso*-17:0 (3-OH), and *iso*-15:0 (2-OH) [4]. Large amounts of long-chain bases are formed, one of which has been identified as dihydrosphingosin [3]. Strain HIM 762-3^T^ contains menaquinone MK-7.

## Genome sequencing and annotation

### Genome project history

This organism was selected for sequencing on the basis of each phylogenetic position, and is part of the *** G****enomic* *** E****ncyclopedia of* *** B****acteria and* *** A****rchaea * project. The genome project is deposited in the Genome OnLine Database [15] and the complete genome sequence in GenBank. Sequencing, finishing and annotation were performed by the DOE Joint Genome Institute (JGI). A summary of the project information is shown in Table 2.

**Table 2:** Genome sequencing project information

MIGS ID	Property	Term
MIGS-31	Finishing quality	Finished
MIGS-28	Libraries used	Two genomic Sanger libraries - 8 kb pMCL200 and fosmid pcc1Fos
MIGS-29	Sequencing platforms	ABI3730
MIGS-31.2	Sequencing coverage	7.5x Sanger
MIGS-30	Assemblers	Phrap
MIGS-32	Gene calling method	Prodigal
	INSDC / Genbank ID	CP001681
	Genbank Date of Release	July 31, 2009
	GOLD ID	Gc01041
	NCBI project ID	27949
	Database: IMG-GEBA	2501533212
MIGS-13	Source material identifier	DSM 2366
	Project relevance	Tree of Life, GEBA

### Growth conditions and DNA isolation

*P. heparinus* strain HIM 762-3^T^, DSM 2366, was grown in DSMZ medium 1 (Nutrient Brot) at 28°C. DNA was isolated from 1-1.5 g of cell paste using Qiagen Genomic 500 DNA Kit (Qiagen, Hilden, Germany) with a modified protocol for cell lysis, adding additonal 100 µl lsozyme; 500 µl chromopeptidase, lysostaphin, mutanolysin, each, to the standard lysis solution, but reducing proteinase K to 160µl, only. Lysis solution was incubated overnight at 35°C on a shaker.

### Genome sequencing and assembly

The genome was sequenced using Sanger sequencing platform only. All general aspects of library construction and sequencing performed at the DOE JGI can be found on their website. The Phred/Phrap/Consed software package was used for sequence assembly and quality assessment. After the shotgun, stage reads were assembled with parallel phrap (High Performance Soft ware, LLC). Possible mis-assemblies were corrected with Dupfinisher [21] or transposon bombing of bridging clones (Epicentre Biotechnologies, Madison, WI). Gaps between contigs were closed by editing in Consed, custom primer walk or PCR amplification (Roche Applied Science, Indianapolis, IN). A total of 1,897 finishing reactions were produced to close gaps and to raise the quality of the finished sequence. The completed genome sequences of *P. heparinus* contains 45,821 Sanger reads, achieving an average of 7.5 x sequence coverage per base with an error rate less than 1 in 100,000.

### Genome annotation

Genes were identified using Prodigal [22] as part of the Oak Ridge National Laboratory genome annotation pipeline, followed by a round of manual curation using the JGI GenePRIMP pipeline. The predicted CDSs were translated and used to search the National Center for Biotechnology Information (NCBI) nonredundant database, UniProt, TIGRFam, Pfam, PRIAM, KEGG, COG, and InterPro databases. Additional gene prediction analysis and functional annotation was performed within the Integrated Microbial Genomes (IMG-ER) platform [23].

### Genome properties

**Figure 3:**
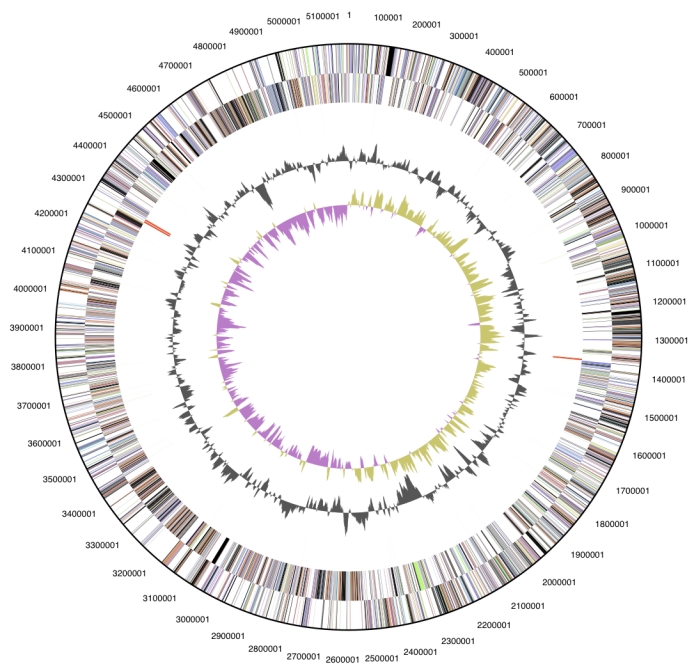
Graphical circular map of the genome. From outside to the center: Genes on forward strand (color by COG categories), Genes on reverse strand (color by COG categories), RNA genes (tRNAs green, rRNAs red, other RNAs black), GC content, GC skew.

The genome is 5,167,383 bp long and comprises one main circular chromosome with a 42.1% GC content (Table 3, Figure 3). Of the 4,341 genes predicted, 4,287 were protein coding genes, and 54 RNAs. Thirty-five pseudogenes were also identified. A minority of the genes (38.1%) were assigned a putative function while the remaining ones were annotated as hypothetical proteins. The properties and the statistics of the genome are summarized in Table 3. The distribution of genes into COGs functional categories is presented in Table 4.

**Table 3:** Genome Statistics

Attribute	Value	% of Total
Genome size (bp)	5,167,383	100.00%
DNA Coding region (bp)	4,829,823	93.47%
DNA G+C content (bp)	2,172,827	42.05%
Number of replicons	1	
Extrachromosomal elements	0	
Total genes	4341	100.00%
RNA genes	54	1.22%
rRNA operons	3	
Protein-coding genes	4287	98.69%
Pseudo genes	35	0.81%
Genes with function prediction	2911	67.05%
Genes in paralog clusters	899	20.70%
Genes assigned to COGs	2806	64.59%
Genes assigned Pfam domains	2991	68.85%
Genes with signal peptides	1425	32.80%
Genes with transmembrane helices	1051	24.19%
CRISPR repeats	0	0.00%

**Table 4:** Number of genes associated with the 21 general COG functional categories

Code	Value	%	Description
J	154	3.6	Translation, ribosomal structure and biogenesis
A	0	0.0	RNA processing and modification
K	281	6.5	Transcription
L	113	2.6	Replication, recombination and repair
B	1	0.0	Chromatin structure and dynamics
D	19	0.4	Cell cycle control, mitosis and meiosis
Y	0	0.0	Nuclear structure
V	59	1.4	Defense mechanisms
T	222	5.2	Signal transduction mechanisms
M	265	6.1	Cell wall/membrane biogenesis
N	13	0.3	Cell motility
Z	0	0.0	Cytoskeleton
W	0	0.0	Extracellular structures
U	48	1.1	Intracellular trafficking and secretion
O	116	2.7	Posttranslational modification, protein turnover, chaperones
C	140	3.3	Energy production and conversion
G	292	6.7	Carbohydrate transport and metabolism
E	209	4.9	Amino acid transport and metabolism
F	65	1.5	Nucleotide transport and metabolism
H	136	3.1	Coenzyme transport and metabolism
I	104	2.4	Lipid transport and metabolism
P	234	5.4	Inorganic ion transport and metabolism
Q	58	1.3	Secondary metabolites biosynthesis, transport and catabolism
R	373	8.7	General function prediction only
S	229	5.3	Function unknown
-	1481	34.5	Not in COGs
